# K-Means Clustering of Hyperpolarised ^13^C-MRI Identifies Intratumoral Perfusion/Metabolism Mismatch in Renal Cell Carcinoma as the Best Predictor of the Highest Grade

**DOI:** 10.3390/cancers17040569

**Published:** 2025-02-07

**Authors:** Ines Horvat-Menih, Alixander S. Khan, Mary A. McLean, Joao Duarte, Eva Serrao, Stephan Ursprung, Joshua D. Kaggie, Andrew B. Gill, Andrew N. Priest, Mireia Crispin-Ortuzar, Anne Y. Warren, Sarah J. Welsh, Thomas J. Mitchell, Grant D. Stewart, Ferdia A. Gallagher

**Affiliations:** 1 Department of Radiology, University of Cambridge, Cambridge CB2 0QQ, UK; ih357@cam.ac.uk (I.H.-M.); ak2290@cam.ac.uk (A.S.K.); mam23@cam.ac.uk (M.A.M.); jd906@medschl.cam.ac.uk (J.D.); e.serrao@nhs.net (E.S.); stephan.ursprung@med.uni-tuebingen.de (S.U.); jk636@cam.ac.uk (J.D.K.); abg28@cam.ac.uk (A.B.G.); anp11@cam.ac.uk (A.N.P.); 2 Department of Radiology, Royal Papworth Hospitals NHS Foundation Trust, Cambridge CB2 0AY, UK; 3 Department of Radiology, Addenbrooke’s Hospital, Cambridge University Hospitals NHS Foundation Trust, Cambridge CB2 0QQ, UK; 4 Department of Oncology, University of Cambridge, Cambridge CB2 0QQ, UK; mc973@cam.ac.uk; 5 Department of Pathology, Addenbrooke’s Hospital, Cambridge University Hospitals NHS Foundation Trust, Cambridge CB2 0QQ, UK; anne.warren@nhs.net; 6 Pinto Medical Consultancy, Cart House 2 Copley Hill Business Park, Cambridge CB22 3GN, UK; sarah.welsh@pintoconsultancy.com; 7 Department of Surgery, University of Cambridge, Cambridge CB2 0QQ, UK; tjm61@cam.ac.uk (T.J.M.); gds35@cam.ac.uk (G.D.S.)

**Keywords:** hyperpolarised [1-^13^C]pyruvate MRI, clear cell renal cell carcinoma, k-means clustering, RNA sequencing

## Abstract

Background: Early and accurate grading of renal cell carcinoma (RCC) improves patient risk stratification and has implications for clinical management and mortality. However, current diagnostic approaches using imaging and renal mass biopsy have limited specificity and may lead to undergrading. Methods: This study explored the use of hyperpolarised [1-^13^C]pyruvate MRI (HP ^13^C-MRI) to identify the most aggressive areas within the tumour of patients with clear cell renal cell carcinoma (ccRCC) as a method to guide biopsy targeting and to reduce undergrading. Six patients with ccRCC underwent presurgical HP ^13^C-MRI and conventional contrast-enhanced MRI. From the imaging data, three k-means clusters were computed by combining the *k*_PL_ as a marker of metabolic activity, and the ^13^C-pyruvate signal-to-noise ratio (SNR_Pyr_) as a perfusion surrogate. The combined clusters were compared to those derived from individual parameters and to those derived from the percentage of enhancement on the nephrographic phase (%NG). The diagnostic performance of each cluster was assessed based on its ability to predict the highest histological tumour grade in postsurgical tissue samples. The postsurgical tissue samples underwent immunohistochemical staining for the pyruvate transporter (monocarboxylate transporter 1, MCT1), as well as RNA and whole-exome sequencing. Results: The clustering approach combining SNR_Pyr_ and *k*_PL_ demonstrated the best performance for predicting the highest tumour grade: specificity 85%; sensitivity 64%; positive predictive value 82%; and negative predictive value 68%. Epithelial MCT1 was identified as the major determinant of the HP ^13^C-MRI signal. The perfusion/metabolism mismatch cluster showed an increased expression of metabolic genes and markers of aggressiveness. Conclusions: This study demonstrates the potential of using HP ^13^C-MRI-derived metabolic clusters to identify intratumoral variations in tumour grade with high specificity. This work supports the use of metabolic imaging to guide biopsies to the most aggressive tumour regions and could potentially reduce sampling error.

## Simple Summary

This study describes a novel way to delineate intratumoral regions within clear cell renal cell carcinoma (ccRCC) based on the clustering of quantitative metabolic images from clinical hyperpolarised [1-^13^C]pyruvate MRI. We show that these clusters, combining metabolic and perfusion metrics, predict the most aggressive tumour region with the highest specificity and outperform clusters derived from standard clinical perfusion imaging. The cluster representing perfusion/metabolism mismatch via imaging showed the highest metabolic dysregulation, with markers of aggressiveness identified through molecular analyses from collected tissue samples. This approach has the potential to guide biopsies to the most aggressive tumour regions, to reduce sampling error and undergrading, as well as to improve risk stratification and clinical management.

## 1. Introduction

Renal cell carcinoma (RCC) represents 2% of cancer diagnoses globally, and an increasing incidence is partly attributed to incidental detection via imaging [1]. However, mortality has not decreased over the last 50 years, despite its earlier detection and improved treatment, with RCC remaining the most lethal urological malignancy [2]. A major diagnostic challenge is differentiating between aggressive small renal masses and indolent lesions, as well as accurately determining tumour grade before surgery [3]. Conventional imaging modalities such as CT and MRI are increasingly used to detect renal masses, but their specificity for determining the presence of ccRCC remains limited [4]. Although a biopsy has a high specificity when a diagnostic sample is acquired, non-diagnostic rates and the potential for undergrading are high (14% and 16%, respectively [5]). While non-invasive diagnostic approaches are highly desirable, there is a lack of blood or urine-based biomarkers in RCC which is attributed to significant intra- and intertumoral heterogeneity [6]. Hence, there is an unmet clinical need for imaging methods to both improve non-invasive diagnosis and to enhance image-guided targeting to the most aggressive intratumoral regions.

Metabolism is particularly useful for phenotyping RCCs, which are characterised by a high degree of metabolic reprogramming, driving tumour formation [7]. For example, in the most common and aggressive RCC subtype, clear cell RCC (ccRCC), the inactivation of the von Hippel-Lindau (*VHL*) gene leads to the stabilisation of hypoxia-inducible factor (HIF) with the downstream activation of angiogenesis and glycolysis and the suppression of the oxidative metabolic pathways [8]. Intratumoral metabolic heterogeneity within RCC has been observed across grades and within spatially separated tumour regions via ex vivo molecular analyses [9,10]. The major clinically available metabolic imaging tool to assess this heterogeneity in vivo is radioactive [^18^F]fluorodeoxyglucose (FDG), detected by positron emission tomography (PET), but this tool has a limited role in renal imaging due to renal excretion of FDG [11]. Hyperpolarised [1-^13^C]pyruvate MRI (HP ^13^C-MRI) is an emerging metabolic imaging technique which involves the injection of a non-radioactive carbon-13 labelled form of pyruvate—an endogenous breakdown product of glucose—which may have a more promising role in renal imaging [12]. Recent studies have shown the potential of the technique in stratifying tumours based on higher World Health Organisation/International Society of Urological Pathology (WHO/ISUP) grade [13,14]. Increased pyruvate metabolism by the enzyme lactate dehydrogenase (LDH), as measured by the resulting lactate formation, correlates with the expression of the pyruvate importer or monocarboxylate transporter 1 (MCT1), which is an independent marker of poor prognosis in ccRCC [14,15].

In this study, we have explored the role of metabolic imaging in assessing tumour aggressiveness by combining metrics of perfusion and metabolism, acquired using HP ^13^C-MRI, to assess whether a combined approach may be more powerful in assessing grade than the individual metrics alone or with conventional contrast-enhanced proton (^1^H) MRI. K-means clustering was used as an unsupervised learning algorithm to cluster pixels based on intensity and spatial positioning to disaggregate areas of metabolic similarity in the tumour, similar to how it has been used previously in ^1^H-MRI to evaluate perfusion in distinct tumour regions [16]. Imaging results were validated on postsurgical tissue samples using MCT1 immunohistochemistry, RNA sequencing (RNAseq) for metabolic pathways and whole-exome sequencing (WES) were performed to determine potential genetic drivers of the imaged signal. The results have shown the potential of metabolic MRI to detect areas of increased tumour aggressiveness, which could be used to guide a biopsy to the most aggressive areas within an individual tumour in the future.

## 2. Materials and Methods

### 2.1. Ethics and Recruitment

Patients were prospectively recruited and provided written informed consent to the following ethically approved studies: Molecular Imaging and Spectroscopy with Stable Isotopes in Oncology and Neurology (MISSION)-Ovary Substudy Renal (ClinicalTrials.gov Identifier NCT03526809) and A translational research Approach to development of optimal Renal cancer Treatments In Surgical and systemic Therapy patients (ARTIST) (NCT04060537). This cohort of patients overlaps with previously published work [14]. Further details are summarised in Supplementary Materials S1, under the section Supplementary Methods (*Ethics and Recruitment*), and in Supplementary Table S1.

### 2.2. MRI Acquisition and Processing

The ^13^C-pyruvate injection and the HP ^13^C-MRI acquisition were performed as described in previously published work and summarised in Supplementary Materials S1, under the section Supplementary Methods (*MRI acquisition and processing*).

^1^H-MRI sequences, including T_1_- and T_2_-weighted (T_1_w, T_2_w) and Gd-contrast-enhanced imaging, were acquired after replacing the ^13^C-tuned coil with the 32-channel cardiac ^1^H-array coil (GE Healthcare, Waukesha WI, USA), and processed as described previously [14]. Percentage nephrographic enhancement (%NG) as a measure of vascular permeability was calculated by subtracting the non-enhanced T_1_-weighted sequence from the nephrographic contrast-enhanced phase, the latter defined as the timepoint of maximal and homogeneous enhancement across renal parenchyma, corresponding to –100 s after the start of Gd injection [17]. To match the imaging planes and correct for any potential movement, the final ^1^H maps were reoriented and manually registered to match the HP ^13^C-MRI using ITK-SNAP 4.0 [18] and in-house-developed MATLAB (MathWorks Inc., Natick MA, USA) scripts. Using the OsiriX Lite 12.0.3 (Pixmeo SARL, Bernex, Switzerland), the regions of interest (ROIs) of whole tumours were drawn on axial T_1_w LavaFlex images by avoiding cystic/necrotic areas via the simultaneous inspection of T_2_w- and Gd-enhanced images.

### 2.3. K-Means Clustering

**Figure 1:**
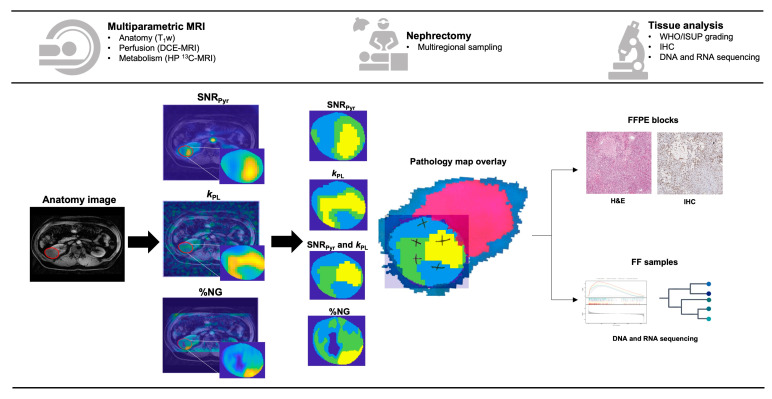
Workflow of the study, as described in Methods. Colour coding of the clusters: lowest mean value = light blue, medium mean value = green, highest mean value = yellow; dark blue regions denoted the background or any voxels with poor signal-to-noise ratio which was matched to background.

ROIs drawn on anatomical T_1_w images were transferred to co-registered HP ^13^C-MRI and %NG images to create masked maps as shown in Figure 1. Masked tumour ROIs were extracted from each map, and clustering was performed using a readily available k-means clustering package as part of the Statistics and Machine Learning Toolbox of the MATLAB software. This was performed on individual HP ^13^C-MRI parameter maps, including SNR_Pyr_ and *k*_PL_, on combined HP ^13^C-MRI maps [SNR_Pyr_ + *k*_PL_], as well as on the %NG map. Outputs of k-means clustering included three clusters, followed by a sorting algorithm to arrange the habitats in order of magnitude to ensure that the highest-numbered cluster always corresponded to the highest magnitude of the imaging data.

### 2.4. Tissue Sampling and Analyses

To enable co-registration of the biopsies to the MRI images, the patient-specific pathology sampling maps were produced from MRI biomarker maps, and a matching 3D-printed tumour mould was created, as described previously [19]. After nephrectomy, each tumour was sliced at a specified location to match the imaging acquisition plane using the tumour mould, and the location of the biopsies was recorded on the patient-specific pathology maps. These were overlaid on top of each cluster as shown in Figure 1, with the normal kidney in purple, perirenal fat in blue, and the location of the biopsies drawn as crosses. Four to fourteen multiregional samples were collected from viable tumour regions by avoiding visible cystic/necrotic areas, and each biopsy was split into half with one part being formalin-fixed paraffin-embedded (FFPE) and the other half flash-frozen. A consultant uropathologist determined the WHO/ISUP grade on H&E of all the biopsies with >75% tumour cellularity. Immunohistochemical (IHC) staining for the MCT1 was performed on the FFPE sections and analysed as described previously [14], while fresh frozen samples underwent RNAseq, WES, and analysis, as described in Supplementary Materials S1.

### 2.5. Statistical Analysis

Statistical analysis was performed in GraphPad Prism v.10 (Dotmatics, Boston MA, USA). All biopsies were assigned to a cluster based on the co-registration of the MRI and the pathology. The diagnostic performance for predicting the highest-grade within the tumour was calculated for all clusters derived from individual and combined clustering. Diagnostic performance was assessed using sensitivity, specificity, and positive and negative predictive values (PPV and NPV), and the area under curve (AUC) of the receiver operating characteristic (ROC) curves served as a consistency measure. MCT1 and cell-type compositions were compared across clusters by applying a one-way analysis of variance (ANOVA) test with Holm–Šidak’s multiple comparison correction for normally distributed data and the Kruskal–Wallis test with Dunn’s multiple comparison correction for nonparametric data, as tested using the Shapiro–Wilk test for normality. Binary comparisons were correspondingly performed using unpaired Student’s *t*-test and the Mann–Whitney U test for gaussian and non-gaussian data, respectively. *p* value < 0.05 was set as a cut-off for statistical significance.

## 3. Results

### 3.1. Study Workflow and Patient Clinical Characteristics

The study workflow is shown in Figure 1, and the summary characteristics of the six patients are presented in Supplementary Table S1. Median age was 59.5 years (range 51–69), and one patient was female. The tumour size ranged from 4.0 to 13.3 cm. Metastatic disease was present in one patient. Median time between imaging and surgery was 16.5 (range 1–119) days. In total, forty-four multiregional samples were post-operatively collected, of which two were excluded due to insufficient tumour cellularity.

### 3.2. K-Means Clustering of HP ^13^C-MRI Detects Intratumoral Heterogeneity of Pyruvate Delivery and Pyruvate-to-Lactate Conversion

**Figure 2:**
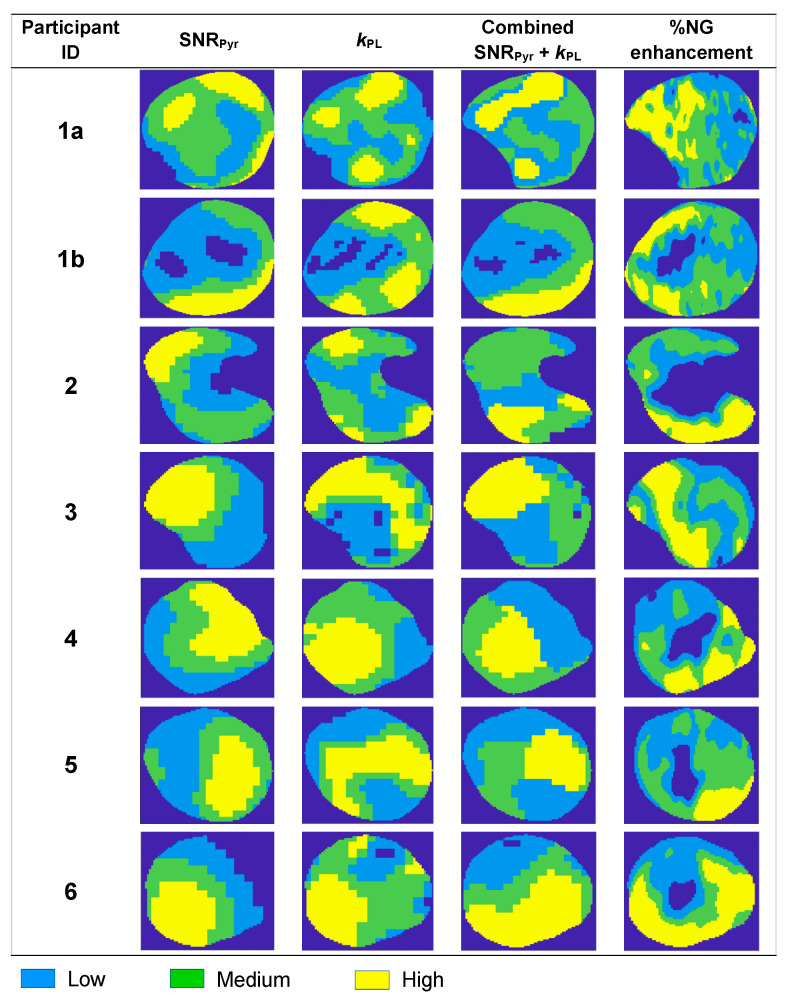
Overview of k-means clustering results in the 6 patients. Tumour from patient 1 was large enough to acquire imaging and biopsies on two slices (1a and 1b), while in other patients, only a single slice was imaged and registered to the post-nephrectomy biopsies. Intratumoral heterogeneity was observed in all clustering approaches.

Figure 2 shows an overview of the clustering results for all patients. Nephrectomy specimens from patients 1 and 5 were sliced at two different axial levels based on the 3D-printed mould which was registered to the HP ^13^C-MRI images as described previously [14]. Slices from patient 1 were sufficiently separated to correspond to two different HP ^13^C-MRI slices (1a and 1b), while the slicing levels of the study patient 5 were both constrained within a single slice on the HP ^13^C-MRI images; thus, biopsies from both levels were overlaid onto the same clustering map. Differences between tissue perfusion (as measured by pyruvate delivery with SNR_Pyr_ and Gd-enhancement with %NG) and tumour metabolism (pyruvate-to-lactate conversion using *k*_PL_) indicated intratumoral heterogeneity in both, which was captured by combining an [SNR_Pyr_ + *k*_PL_] clustering approach.

The normalised individual and combined SNR_Pyr_ and *k*_PL_ means within each combined cluster group were compared in order to quantify the level of heterogeneity of perfusion and metabolism within and between the combined clusters (Supplementary Figure S1).

The medium cluster showed the greatest degree of perfusion/metabolism mismatch with low perfusion and high metabolic conversion, which may represent increased glycolytic metabolism secondary to tumour hypoxia (medium cluster mean *k*_PL_/SNR_Pyr_ ratio = 1.4, compared to 1.0 and 0.9 in the low and high combined clusters, respectively; Supplementary Figure S1A). This is analogous to discrepancies found through ^18^F-FDG-PET imaging, which have been reported as a feature of tumour aggressiveness [20,21,22].

Supplementary Figure S1B shows values of individual SNR_Pyr_, *k*_PL_, and combined [SNR_Pyr_ + *k*_PL_] across combined [SNR_Pyr_ + *k*_PL_] clusters. SNR_Pyr_ was significantly different in the low vs. high (Dunn’s multiple comparison test adjusted *p* < 0.01) and medium vs. high (*p* < 0.001) clusters, but not in the low vs. medium combined clusters (*p* > 0.99). *k*_PL_ across combined clusters was significantly different in the low vs. medium (*p* < 0.01) and low vs. high (*p* < 0.0001) clusters, but not between medium vs. high clusters (*p* = 0.20). The normalised mean of the combined [SNR_Pyr_ + *k*_PL_] metric increased from the low to the high cluster and showed a significant difference in the low vs. high (*p* < 0.0001) and medium vs. high (*p* < 0.001) comparison, but not between the low vs. medium clusters (*p* = 0.06).

Altogether, these results suggest significant inter- and intra-cluster variations in perfusion and metabolism.

### 3.3. The Cluster with the Greatest Perfusion/Metabolism Mismatch Predicts the Highest Intratumoral Grade

The diagnostic performance of each clustering approach in predicting the highest-grade intratumoral region, by comparing these results to clusters derived from %NG enhancement, was assessed in order to explore the hypothesis that perfusion/metabolism mismatch may correspond to the most aggressive tumour regions. The diagnostic performance metrics of all the clustering approaches for detecting the highest grade within the tumour are shown in Table 1 intratumoral variation in grades or investigated metabolism beyond pyruvate-to-lactate conversion. ROC curves are presented in Supplementary Figure S2. Calculations are shown in Supplementary Tables S2 and S3.

The medium combined [SNR_Pyr_ + *k*_PL_] cluster exhibited the highest diagnostic performance rates amongst all the clustered parameters: specificity 85%; sensitivity 64%; PPV 82%; and NPV 68%. Although specificity was comparable to the high SNR_Pyr_ cluster and the sensitivity to the medium %NG cluster, the combined clustering showed the highest consistency, as suggested via the ROC curve analysis (AUC 0.67). Therefore, the medium combined [SNR_Pyr_ + *k*_PL_] cluster, reflecting the low pyruvate delivery and high metabolic conversion (Supplementary Figure S1A), demonstrated the best predictor of the highest-grade within the tumour. This prompted further exploration into molecular processes underlying HP ^13^C-MRI clustering.

### 3.4. The Epithelial Compartment Is the Predominant Cell Type and Demonstrates High Pyruvate Transporter Expression, Suggesting a Significant Role in HP ^13^C-MRI Signal Generation

**Figure 3:**
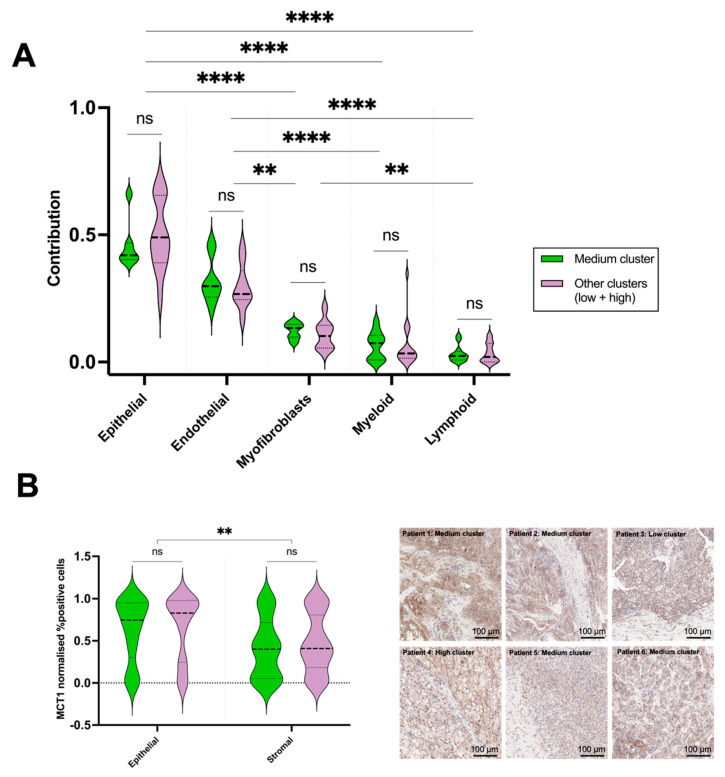
Comparison of tumour microenvironment characteristics within combined clusters. The results were normalised by linear scaling with plots representing (**A**) deconvoluted RNAseq-identifying cell-type-specific signatures and (**B**) Comparison of MCT1 expression in epithelial and stromal compartments, with representative IHC images from each patient on the right-hand side. ** = *p* < 0.01; **** = *p* < 0.0001.

A possible contribution of the tumour microenvironment (TME) and the pyruvate transporter MCT1 to the generation of combined clusters was analysed by deconvoluting the bulk RNAseq for cell-type-specific signatures and by quantifying the epithelial and stromal MCT1 staining. Results were compared in binary fashion where the medium combined cluster was compared to the others (low + high), as presented in Figure 3.

Deconvoluted RNAseq-derived cell specific signatures revealed that tumour epithelium contributed the largest relative cellular contribution in the samples, followed by the endothelial component. Both compartments exhibited significantly higher contributions compared to other compartments, as determined by Dunn’s multiple comparison test (Figure 3A). However, no difference in cell composition was observed in the comparisons between the medium combined [SNR_Pyr_ + *k*_PL_] cluster and the others.

MCT1 expression was significantly higher in the epithelial compartment compared to the stromal portion (Figure 3B), but no significant differences were found in the intra-cluster comparison. As MCT1 is the main transporter for pyruvate uptake, this is consistent with the epithelial compartment being a major determinant of the HP ^13^C-MRI signal, similar to what has been demonstrated in other tumour types such as prostate cancer [23]. Taken together, these results support the hypothesis that the HP ^13^C-MRI signal may be weighted towards metabolism in the epithelial tumour compartment, but between the clusters, no TME differences were identified.

### 3.5. The Medium Combined Cluster Revealed the Highest Metabolic Gene Expression and Aggressive Tumour Signatures Based on the Transcriptomic Analysis

**Figure 4:**
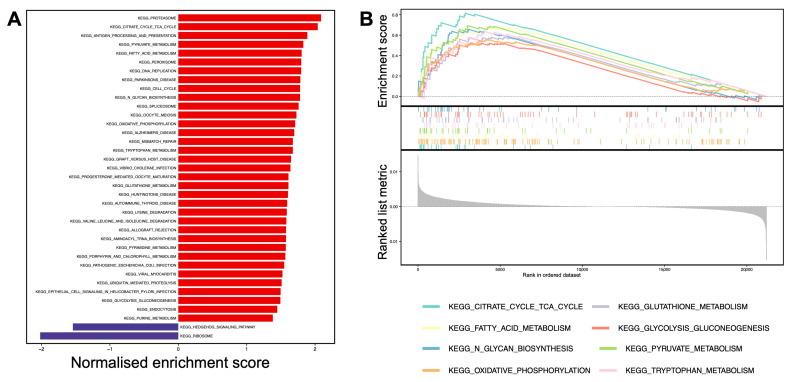
Transcriptomic gene score enrichment analysis for the KEGG MSigDB-curated gene set. (**A**) Barplots and (**B**) classic GSEA plots of combined [SNR_Pyr_ + *k*_PL_] clustering, comparing the medium cluster to the others (low + high). Red and blue bars represent up- and down-regulated pathways in the medium cluster compared to the others, respectively.

To understand the metabolic dysregulation underlying the combined [SNR_Pyr_ + *k*_PL_] clusters, GSEA was performed using KEGG MSigDB gene sets. Figure 4 displays pathways with significance as defined by Benjamini–Hochberg *p*-adjusted values < 0.05 based on the comparison of the medium combined cluster to the others (low + high). Supplementary Materials S2 contains detailed statistical results.

The medium combined cluster exhibited upregulated metabolic signatures compared to the (low + high) combined clusters together. Notably, the energy-yielding metabolic pathways including the tricarboxylic acid (TCA) cycle, oxidative phosphorylation (OXPHOS), and pyruvate metabolism ranked among the highest normalised enrichment scores. This was associated with upregulated glutathione and peroxisomal pathways, indicating changes in oxidative stress and redox homeostasis. Other significantly enriched metabolic pathways included fatty acid metabolism, N-glycan biosynthesis, glycolysis and gluconeogenesis, and tryptophan metabolism. The upregulation of the proteasome and valine–leucine–isoleucine degradation pathways suggested increased peptide turnover, which may be explained by enhanced proliferation and the requirement for biosynthetic material. In support of this, cell cycle, DNA replication (increased pyrimidine and purine metabolism), and mismatch repair mechanisms were upregulated. The antigen processing and presentation (APP) pathway was also upregulated in the medium cluster compared to the others.

### 3.6. Genetic Divergence May Partly Underlie the Aggressive Phenotype

**Figure 5:**
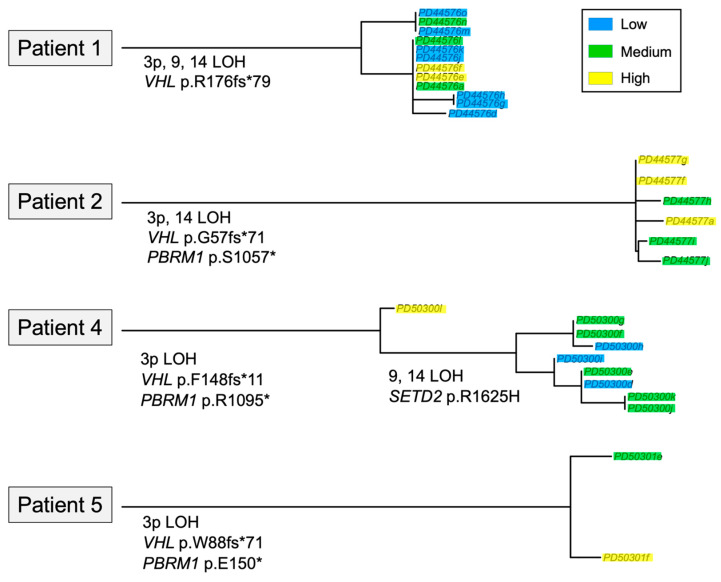
Phylogenies of the genetic alterations in 4 patients. Legend on the top right corner describes the colour-coding: blue = low cluster, green = medium cluster, and yellow = high cluster. Only in this figure; * denotes position of the stop codon.

The genetic drivers of ccRCC are well-established, with *VHL* loss found in 75% of cases, leading to downstream metabolic phenotype perturbations [24]. To examine the impact of genetic drivers in differentiating the imaging clusters derived from the combined approach, we performed genetic phylogeny analysis, as depicted in Figure 5. Tissue samples from patients 3 and 6 failed to meet the WES quality control criteria, limiting analysis to the remaining patients.

*VHL* loss was identified as the truncal mutation in all four patients, indicating that it is not a causative factor for intratumoral metabolic heterogeneity. Similarly, 3p loss of heterozygosity (LOH) was detected in all patients, and most other genetic alterations were truncal in origin, such as 14q LOH in patient 1 and *PBRM1* alterations in patients 2, 4, and 5. The only exception was the early branching of 9, 14 LOH, and the *SETD2* arginine-to-histidine substitution in patient 4, which was notably detected in all samples from the combined [SNR_Pyr_ + *k*_PL_] medium clusters.

## 4. Discussion

Here, we have employed HP ^13^C-MRI combined with k-means clustering analysis to investigate whether regional changes in metabolism across renal tumours can be used to detect the most aggressive intratumoral regions, with the ultimate aim of guiding biopsy procedures in the future.

To the best of our knowledge, this is the first report of clustering on clinical HP ^13^C-MRI data to define intratumoral metabolic habitats in order to assess their ability to detect the highest ccRCC grade within the tumour [25]. In particular, combining the metrics for pyruvate delivery and metabolic conversion to lactate [SNR_Pyr_ + *k*_PL_] demonstrated the highest diagnostic performance for predicting tumour grade, and this method was superior to the clustering approach where each component was used separately or compared to conventional contrast-enhanced MRI. The medium combined cluster showed the highest sensitivity for the detection of aggressive disease and the greatest disparity between metabolism and perfusion. Such perfusion/metabolism mismatch as a feature of tumour aggressiveness is analogous to that reported previously via FDG-PET imaging [20], but these studies either employed different probes to separately determine blood flow and glucose uptake (e.g., ^15^O-H_2_O and ^18^F-FDG respectively reported in pancreatic [26], breast [21], and cervical cancer [22]) or different modalities (e.g., contrast-enhanced CT with FDG-PET in oesophageal cancer [27]). Here, we have used a single injection of hyperpolarised ^13^C-pyruvate and HP ^13^C-MRI to extract these parameters within seconds of injection and in the absence of ionising radiation, which is of particular importance in imaging of the kidneys due to the renal excretion of ^18^F-FDG, which complicates any interpretation [11]. The investigation of perfusion/metabolism mismatch using HP ^13^C-MRI has been reported preclinically using a dual contrast agent approach (^13^C-pyruvate and ^13^C-urea) in a murine prostate cancer model which found a correlation between decreased perfusion and increased metabolism with higher intertumoral grades [28].

It has been previously reported that imaging metrics of pyruvate-to-lactate conversion, such as *k*_PL_ and the LAC/PYR ratio, positively correlate with higher renal tumour aggressiveness [13,14]. Conventional proton MRI metrics of perfusion have been used to detect higher ccRCC grades; for example, lower arterial spin labelling (ASL)-measured perfusion has been reported in higher ccRCC grades [29], consistent with lower microvascular density as assessed using IHC. However, *K*^trans^ as a measure of vascularity via DCE-MRI has shown variability between and within tumours, suggesting inter- and intratumoral heterogeneity [29]. *K*^trans^ is not a pure measure of perfusion but reflects the inflow of the contrast agent from large vessels and its exchange rate into the interstitial space, therefore incorporating a measure of tumour vascular permeability [30]. Here, we show that the high cluster of the vascularity parameter (%NG) had the best specificity to predict higher grade region within the tumour, while other metrics varied in their performance. Similarly, Xi et al. reported that the high Gd-enhancing k-means cluster within a tumour is most predictive of a higher grade across a cohort of 18 patients with ccRCC [31]; however, these results relied on a single postsurgical grade for the whole tumour rather than examining the grade variation across the entire tumour. Udayakumar et al. correlated the intratumoral variation in early nephrogenic enhancement with angiogenic and immune transcriptomic signatures of biopsies from the same regions [32], and Yao et al. reported two types of microvessels which are differentially expressed between low- and high-grade ccRCCs, indicating the complexity of ccRCC perfusion [33]. These results indicate that tumour perfusion is an important determinant of aggressiveness, but when used alone, it is insufficient to fully and accurately characterise the tumour grade non-invasively. Therefore, combining perfusion with other biological processes such as metabolism, as we have undertaken here, has the potential to improve prediction.

Although intratumoral heterogeneity of pyruvate metabolism in ccRCC has been studied before, this work has extended this by evaluating the predictive power of HP ^13^C-MRI-derived metabolic clusters and by investigating the underlying biology beyond pyruvate, including cellular compartmentalisation and transcriptomic and genomic signatures. The epithelial cell compartment exhibited the highest expression of the pyruvate importer MCT1 and may be the driving factor for the generation of the HP ^13^C-MRI signal as previously suggested [23,34], although no differences were found between the clusters. Thus, the intratumoral clusters were attributed to variations in metabolic dysregulation as identified via our transcriptomic analysis. This is supported by Okegawa et al. [10] who identified differential pyruvate profiles across spatially separated tumour biopsies of eight ccRCC patients, and the high pyruvate cluster was matched with low *LDHA* expression. Through isotope tracing experiments, they found higher PDH flux in certain regions but have not investigated how this relates to tumour grade or the signal via imaging. Hakimi et al. [35] and Li et al. [36] have identified how ccRCC metabolic clusters are linked to tumour stage and to survival outcomes, but they have only addressed intertumoral rather than intratumoral heterogeneity. Applying HP ^13^C-MRI, Tran et al. [37] reported the highest lactate level, determined via mass spectrometry (MS), corresponding to the highest ^13^C-lactate signal within a single ccRCC, but they did not investigate the intratumoral variation in grades or investigated metabolism beyond pyruvate-to-lactate conversion.

Through RNAseq analysis, we identified upregulated metabolic pathways in the medium combined [SNR_Pyr_ + *k*_PL_] cluster, particularly the notably upregulated TCA cycle pathway. Bezwada et al. have previously detected enhanced labelling of TCA intermediates in ccRCC metastases compared to the primary tumour during isotope labelling experiments in patients, suggesting that the harnessing of oxidative pathways provides a selective advantage for ccRCC progression [38]. This was further linked to the maintenance of redox balance, which contributes to aggressiveness by enabling the survival of tumour cells [39]. In our study, the antioxidant-sustaining pathways including the glutathione and peroxisome pathways were highly upregulated. Glutathione acts as a scavenger for reactive oxygen species (ROS) to sustain malignant growth [40], and peroxisomes are also known to regulate lipid droplet formation, which is the defining histological feature of ccRCC [41]. The storage of these increased fatty acids is necessary to suppress lipotoxicity and to maintain cell membranes in ccRCC [41]. Glycogen is known to accumulate in these droplets, altering protein glycosylation [42]. Such abnormal glycosylation was shown to promote cell–cell signalling, invasion, and migration [43], and we have shown increased N-glycan biosynthesis in the most aggressive combined cluster in this study. The tryptophan metabolic pathway was the most prominently upregulated among the amino acid pathways, which is well described in ccRCC for its immunosuppressive effects via the kynurenine pathway [44]. In the medium combined [SNR_Pyr_ + *k*_PL_] cluster, the cell proliferation pathways were upregulated, which is tightly linked to higher cancer aggressiveness and used in clinical routine histological grading of ccRCC based on nucleolar prominence [45].

This study is the first to compare gene aberrations to metabolic phenotypes detected using HP ^13^C-MRI in ccRCC. The intratumoral genomic clonality of ccRCC has been extensively studied [46,47], but it remains unclear how this translates to a variation in metabolic phenotypes, but evidence suggests that it is only partly related to genetic alterations [48]. We identified truncal *VHL* loss in all the patients, and although this is a major driver of the difference in metabolism between tumour and normal tissue, it is unlikely to contribute to the intratumoral metabolic heterogeneity detected using HP ^13^C-MRI. This finding was consistent with Okegawa et al. [10], who reported intratumoral variation in pyruvate-to-lactate conversion via mass spectrometry that was independent of *VHL* gene status. While most other genetic alterations were truncal, the single exception of genetic branching was the LOH of chromosomes 9 and 14 and the *SETD2* mutation in patient 4, which were all found within the combined medium cluster. LOH of chromosomes 9 and 14, as well as elevated genomic intratumoral heterogeneity, has been previously associated with more adverse outcomes [49,50,51], and this supports our finding that the medium combined cluster represents the most aggressive intratumoral region. In addition, *SETD2* loss has been previously found to promote ccRCC expansion through replication stress and defective DNA damage repair [52], as well as a switch of ccRCC metabolism towards OXPHOS [53], in line with our finding of upregulated OXPHOS pathway in the medium combined cluster. Although this was the first study to link intratumoral genomic variations with metabolic clusters via imaging, no direct causation was observed due to the limited number of patients. Future studies will need to further elucidate the extent to which additional factors, such as epigenetics and noncanonical metabolic flux, regulate metabolic phenotypes [54].

Our research faced unavoidable technical challenges: HP ^13^C-MRI images have inherently low resolution (voxel size: 17 × 17 × 30 mm^3^), which may dilute the border between heterogeneous regions, especially in the superior–inferior direction. This was the reason for allocating biopsies from two different axial levels of tissue sampling onto the same HP ^13^C-image for patient 5 in the study. Therefore, only the 2D analysis of HP ^13^C-MRI was possible. Also, biopsy sampling was performed only on slices of nephrectomised kidneys since whole-tumour tissue analyses are technically challenging even for research purposes and impossible in a clinical setting due to the need for diagnostic samples. However, multiregional sampling with the 3D-printed tumour mould enabled a good correspondence between imaging and pathology [19], and by clustering ^13^C-data, we mitigated the effects of any possible misregistration, thereby providing a larger confidence space for potential future biopsy targeting. Finally, it is important to state that while the findings of this study appear promising for the clinical management of RCC, the feasibility of implementing HP ^13^C-MRI with subsequent k-means clustering analysis currently presents a challenge due to the high cost and technical requirements. However, this research field is rapidly advancing, and further innovations may enable the clinical integration of HP ^13^C-MRI in the near future [55].

## 5. Conclusions

In summary, we present a novel clustering method of HP ^13^C-MRI data that predicts the most aggressive intratumoral ccRCC grades with high specificity, outperforming individual parameters and the clinical standard. This work supports the potential of metabolic imaging to guide a biopsy to the most aggressive tumour region, thereby reducing sampling error and undergrading, improving both risk stratification and ultimately clinical management strategies. Additionally, the analysis of underlying molecular processes sets the ground for future research into the intratumoral metabolic heterogeneity of ccRCC with potential for the development of novel treatments.

## Figures and Tables

**Table 1:** Comparison of the diagnostic performance of the k-means clustering approaches for the detection of the highest grade within an individual tumour. PPV = positive predictive value, NPV = negative predictive value, AUC = area under the curve.

Clustering Approach	Sensitivity	Specificity	PPV	NPV	AUC
**SNR_Pyr_ only**					
Low	40%	70%	60%	52%	0.58
Medium	46%	50%	50%	46%	0.58
High	23%	85%	63%	50%	0.54
***k*_PL_ only**					
Low	18%	70%	40%	44%	0.58
Medium	55%	80%	75%	62%	0.58
High	36%	55%	47%	44%	0.59
**Combined SNR_Pyr_ + *k*_PL_**					
Low	32%	65%	50%	46%	0.56
Medium	64%	85%	82%	68%	0.67
High	14%	55%	25%	37%	0.50
**%Nephrographic enhancement**					
Low	27%	60%	43%	43%	0.60
Medium	64%	65%	67%	62%	0.60
High	18%	80%	50%	47%	0.57

## Data Availability

All data that support the findings of this study are available upon reasonable request from the corresponding author, on the condition that this will not be used to deanonymize the patients. The data are not publicly available due to them containing information that could compromise research participant privacy and consent.

## Supplementary Materials

The following are available online at https://www.mdpi.com/article/10.3390/cancers17040569/s1, Supplementary Materials S1 (Supplementary Methods and Supplementary Tables and Figures) [14,56,57,58,59,60,61,62,63,64,65,66,67,68,69,70,71]; Supplementary Materials S2.

## References

[1] Padala S.A., Barsouk A., Thandra K.C., Saginala K., Mohammed A., Vakiti A., Rawla P., Barsouk A. (2020). Epidemiology of Renal Cell Carcinoma. World J. Oncol..

[2] Kratzer T.B., Siegel R.L., Miller K.D., Sung H., Islami F., Jemal A. (2022). Progress Against Cancer Mortality 50 Years After Passage of the National Cancer Act. JAMA Oncol..

[3] Kay F.U., Pedrosa I. (2018). Imaging of Solid Renal Masses. Urol. Clin. N. Am..

[4] Yang Z., Li M., Guo A., Liang Y., Fang P. (2022). Imaging Features and Clinic Value of Mri and Ct in Diagnosis of Clear Cell Renal Cell Carcinoma. Food Sci. Technol..

[5] Patel H.D., Johnson M.H., Pierorazio P.M., Sozio S.M., Sharma R., Iyoha E., Bass E.B., Allaf M.E. (2016). Diagnostic Accuracy and Risks of Biopsy in the Diagnosis of a Renal Mass Suspicious for Localized Renal Cell Carcinoma: Systematic Review of the Literature. J. Urol..

[6] Bex A., Albiges L., Bedke J., Bonn S., Capitanio U., Dabestani S., Hora M., Klatte T., Kuusk T., Lund L. EAU Guidelines: Renal Cell Carcinoma. https://uroweb.org/guideline/renal-cell-carcinoma/.

[7] Chakraborty S., Balan M., Sabarwal A., Choueiri T.K., Pal S. (2021). Metabolic Reprogramming in Renal Cancer: Events of a Metabolic Disease. Biochim. Biophys. Acta.

[8] Yong C., Stewart G.D., Frezza C. (2020). Oncometabolites in Renal Cancer. Nat. Rev. Nephrol..

[9] Wettersten H.I., Hakimi A.A., Morin D., Bianchi C., Johnstone M.E., Donohoe D.R., Trott J.F., Aboud O.A., Stirdivant S., Neri B. (2015). Grade-Dependent Metabolic Reprogramming in Kidney Cancer Revealed by Combined Proteomics and Metabolomics Analysis. Cancer Res..

[10] Okegawa T., Morimoto M., Nishizawa S., Kitazawa S., Honda K., Araki H., Tamura T., Ando A., Satomi Y., Nutahara K. (2017). Intratumor Heterogeneity in Primary Kidney Cancer Revealed by Metabolic Profiling of Multiple Spatially Separated Samples within Tumors. EBioMedicine.

[11] Karivedu V., Jain A.L., Eluvathingal T.J., Sidana A. (2019). Role of Positron Emission Tomography Imaging in Metabolically Active Renal Cell Carcinoma. Curr. Urol. Rep..

[12] Vaeggemose M., Schulte R.F., Laustsen C. (2021). Comprehensive Literature Review of Hyperpolarized Carbon-13 MRI: The Road to Clinical Application. Metabolites.

[13] Tang S., Meng M.V., Slater J.B., Gordon J.W., Vigneron D.B., Stohr B.A., Larson P.E.Z., Wang Z.J. (2021). Metabolic Imaging with Hyperpolarized ^13^C Pyruvate Magnetic Resonance Imaging in Patients with Renal Tumors—Initial Experience. Cancer.

[14] Ursprung S., Woitek R., McLean M.A., Priest A.N., Crispin-Ortuzar M., Brodie C.R., Gill A.B., Gehrung M., Beer L., Riddick A.C.P. (2022). Hyperpolarized 13C-Pyruvate Metabolism as a Surrogate for Tumor Grade and Poor Outcome in Renal Cell Carcinoma—A Proof of Principle Study. Cancers.

[15] Cao Y.-W., Liu Y., Dong Z., Guo L., Kang E.-H., Wang Y.-H., Zhang W., Niu H.-T. (2018). Monocarboxylate Transporters MCT1 and MCT4 Are Independent Prognostic Biomarkers for the Survival of Patients with Clear Cell Renal Cell Carcinoma and Those Receiving Therapy Targeting Angiogenesis. Urol. Oncol. Semin. Orig. Investig..

[16] Jardim-Perassi B.V., Huang S., Dominguez-Viqueira W., Poleszczuk J., Budzevich M.M., Abdalah M.A., Pillai S.R., Ruiz E., Bui M.M., Zuccari D.A. (2019). Multiparametric MRI and Coregistered Histology Identify Tumor Habitats in Breast Cancer Mouse Models. Cancer Res..

[17] Wang Z.J., Westphalen A.C., Zagoria R.J. (2018). CT and MRI of Small Renal Masses. Br. J. Radiol..

[18] Yushkevich P.A., Piven J., Hazlett H.C., Smith R.G., Ho S., Gee J.C., Gerig G. (2006). User-Guided 3D Active Contour Segmentation of Anatomical Structures: Significantly Improved Efficiency and Reliability. NeuroImage.

[19] Crispin-Ortuzar M., Gehrung M., Ursprung S., Gill A.B., Warren A.Y., Beer L., Gallagher F.A., Mitchell T.J., Mendichovszky I.A., Priest A.N. (2020). Three-Dimensional Printed Molds for Image-Guided Surgical Biopsies: An Open Source Computational Platform. JCO Clin. Cancer Inform..

[20] Mankoff D.A., Dunnwald L.K., Partridge S.C., Specht J.M. (2009). Blood Flow-Metabolism Mismatch: Good for the Tumor, Bad for the Patient. Clin. Cancer Res..

[21] Specht J.M., Kurland B.F., Montgomery S.K., Dunnwald L.K., Doot R.K., Gralow J.R., Ellis G.K., Linden H.M., Livingston R.B., Allison K.H. (2010). Tumor Metabolism and Blood Flow as Assessed by Positron Emission Tomography Varies by Tumor Subtype in Locally Advanced Breast Cancer. Clin. Cancer Res..

[22] Apostolova I., Hofheinz F., Buchert R., Steffen I.G., Michel R., Rosner C., Prasad V., Köhler C., Derlin T., Brenner W. (2014). Combined Measurement of Tumor Perfusion and Glucose Metabolism for Improved Tumor Characterization in Advanced Cervical Carcinoma: A PET/CT Pilot Study Using [^15^O]Water and [^18^F]Fluorodeoxyglucose. Strahlenther. Onkol..

[23] Sushentsev N., McLean M.A., Warren A.Y., Benjamin A.J.V., Brodie C., Frary A., Gill A.B., Jones J., Kaggie J.D., Lamb B.W. (2022). Hyperpolarised ^13^C-MRI Identifies the Emergence of a Glycolytic Cell Population within Intermediate-Risk Human Prostate Cancer. Nat. Commun..

[24] Gossage L., Murtaza M., Slatter A.F., Lichtenstein C.P., Warren A., Haynes B., Marass F., Roberts I., Shanahan S.J., Claas A. (2014). Clinical and Pathological Impact of *VHL, PBRM1, BAP1, SETD2, KDM6A*, and *JARID1c* in Clear Cell Renal Cell Carcinoma: VHL, PBRM1, BAP1, JARID1c, SETD2, & KDM6a in CCRCC. Genes Chromosomes Cancer.

[25] Daniels C.J., Gallagher F.A. (2018). Unsupervised Segmentation of 5D Hyperpolarized Carbon-13 MRI Data Using a Fuzzy Markov Random Field Model. IEEE Trans. Med. Imaging.

[26] Komar G., Kauhanen S., Liukko K., Seppänen M., Kajander S., Ovaska J., Nuutila P., Minn H. (2009). Decreased Blood Flow with Increased Metabolic Activity: A Novel Sign of Pancreatic Tumor Aggressiveness. Clin. Cancer Res..

[27] Zhao K., Wang C., Mao Q., Shang D., Huang Y., Ma L., Yu J., Li M. (2020). The Flow-Metabolism Ratio Might Predict Treatment Response and Survival in Patients with Locally Advanced Esophageal Squamous Cell Carcinoma. EJNMMI Res..

[28] Chen H.-Y., Larson P.E.Z., Bok R.A., von Morze C., Sriram R., Delos Santos R., Delos Santos J., Gordon J.W., Bahrami N., Ferrone M. (2017). Assessing Prostate Cancer Aggressiveness with Hyperpolarized Dual-Agent 3D Dynamic Imaging of Metabolism and Perfusion. Cancer Res..

[29] Zhang Y., Kapur P., Yuan Q., Xi Y., Carvo I., Signoretti S., Dimitrov I., Cadeddu J.A., Margulis V., Muradyan N. (2016). Tumor Vascularity in Renal Masses: Correlation of Arterial Spin-Labeled and Dynamic Contrast-Enhanced Magnetic Resonance Imaging Assessments. Clin. Genitourin. Cancer.

[30] Profiles—QIBA Wiki. https://qibawiki.rsna.org/index.php/Profiles.

[31] Xi Y., Yuan Q., Zhang Y., Madhuranthakam A.J., Fulkerson M., Margulis V., Brugarolas J., Kapur P., Cadeddu J.A., Pedrosa I. (2018). Statistical Clustering of Parametric Maps from Dynamic Contrast Enhanced MRI and an Associated Decision Tree Model for Non-Invasive Tumour Grading of T1b Solid Clear Cell Renal Cell Carcinoma. Eur. Radiol..

[32] Udayakumar D., Zhang Z., Xi Y., Dwivedi D.K., Fulkerson M., Haldeman S., McKenzie T., Yousuf Q., Joyce A., Hajibeigi A. (2021). Deciphering Intratumoral Molecular Heterogeneity in Clear Cell Renal Cell Carcinoma with a Radiogenomics Platform. Clin. Cancer Res..

[33] Yao X., Qian C.-N., Zhang Z.-F., Tan M.-H., Kort E.J., Yang X.J., Resau J.H., Teh B.T. (2007). Two Distinct Types of Blood Vessels in Clear Cell Renal Cell Carcinoma Have Contrasting Prognostic Implications. Clin. Cancer Res..

[34] Rao Y., Gammon S., Zacharias N.M., Liu T., Salzillo T., Xi Y., Wang J., Bhattacharya P., Piwnica-Worms D. (2020). Hyperpolarized [1-^13^C]Pyruvate-to-[1-^13^C]Lactate Conversion Is Rate-Limited by Monocarboxylate Transporter-1 in the Plasma Membrane. Proc. Natl. Acad. Sci. USA.

[35] Hakimi A.A., Reznik E., Lee C.-H., Creighton C.J., Brannon A.R., Luna A., Aksoy B.A., Liu E.M., Shen R., Lee W. (2016). An Integrated Metabolic Atlas of Clear Cell Renal Cell Carcinoma. Cancer Cell.

[36] Li L., Chao Z., Waikeong U., Xiao J., Ge Y., Wang Y., Xiong Z., Ma S., Wang Z., Hu Z. (2023). Metabolic Classifications of Renal Cell Carcinoma Reveal Intrinsic Connections with Clinical and Immune Characteristics. J. Transl. Med..

[37] Tran M., Latifoltojar A., Neves J.B., Papoutsaki M.-V., Gong F., Comment A., Costa A.S.H., Glaser M., Tran-Dang M.-A., Sheikh S.E. (2019). First-in-Human in Vivo Non-Invasive Assessment of Intra-Tumoral Metabolic Heterogeneity in Renal Cell Carcinoma. BJR Case Rep..

[38] Bezwada D., Lesner N.P., Brooks B., Vu H.S., Wu Z., Cai L., Kasitinon S., Kelekar S., Cai F., Aurora A.B. (2023). Mitochondrial Metabolism in Primary and Metastatic Human Kidney Cancers. bioRxiv.

[39] Purohit V., Simeone D.M., Lyssiotis C.A. (2019). Metabolic Regulation of Redox Balance in Cancer. Cancers.

[40] Xiao Y., Meierhofer D. (2019). Glutathione Metabolism in Renal Cell Carcinoma Progression and Implications for Therapies. IJMS.

[41] Tan S.K., Hougen H.Y., Merchan J.R., Gonzalgo M.L., Welford S.M. (2023). Fatty Acid Metabolism Reprogramming in ccRCC: Mechanisms and Potential Targets. Nat. Rev. Urol..

[42] Drake R.R., McDowell C., West C., David F., Powers T.W., Nowling T., Bruner E., Mehta A.S., Angel P.M., Marlow L.A. (2020). Defining the Human Kidney N-Glycome in Normal and Cancer Tissues Using MALDI Imaging Mass Spectrometry. J. Mass Spectrom..

[43] Zhu X., Al-Danakh A., Zhang L., Sun X., Jian Y., Wu H., Feng D., Wang S., Yang D. (2022). Glycosylation in Renal Cell Carcinoma: Mechanisms and Clinical Implications. Cells.

[44] Trott J.F., Kim J., Aboud O.A., Wettersten H., Stewart B., Berryhill G., Uzal F., Hovey R.C., Chen C.-H., Anderson K. (2016). Inhibiting Tryptophan Metabolism Enhances Interferon Therapy in Kidney Cancer. Oncotarget.

[45] Delahunt B., Eble J.N., Egevad L., Samaratunga H. (2019). Grading of Renal Cell Carcinoma. Histopathology.

[46] Gerlinger M., Horswell S., Larkin J., Rowan A.J., Salm M.P., Varela I., Fisher R., McGranahan N., Matthews N., Santos C.R. (2014). Genomic Architecture and Evolution of Clear Cell Renal Cell Carcinomas Defined by Multiregion Sequencing. Nat. Genet..

[47] Patel S.A., Rodrigues P., Wesolowski L., Vanharanta S. (2020). Genomic Control of Metastasis. Br. J. Cancer.

[48] Le A. (2021). The Heterogeneity of Cancer Metabolism, Advances in Experimental Medicine and Biology.

[49] Nejati R., Wei S., Uzzo R.G., Poureghbali S., Pei J., Talarchek J.N., Ruth K., Dulaimi E., Kutikov A., Testa J.R. (2020). Monosomy of Chromosome 9 Is Associated With Higher Grade, Advanced Stage, and Adverse Outcome in Clear-Cell Renal Cell Carcinoma. Clin. Genitourin. Cancer.

[50] Monzon F.A., Alvarez K., Peterson L., Truong L., Amato R.J., Hernandez-McClain J., Tannir N., Parwani A.V., Jonasch E. (2011). Chromosome 14q Loss Defines a Molecular Subtype of Clear-Cell Renal Cell Carcinoma Associated with Poor Prognosis. Mod. Pathol..

[51] Turajlic S., Xu H., Litchfield K., Rowan A., Horswell S., Chambers T., O’Brien T., Lopez J.I., Watkins T.B.K., Nicol D. (2018). Deterministic Evolutionary Trajectories Influence Primary Tumor Growth: TRACERx Renal. Cell.

[52] Kanu N., Grönroos E., Martinez P., Burrell R.A., Goh X.Y., Bartkova J., Maya-Mendoza A., Mistrík M., Rowan A.J., Patel H. (2015). SETD2 Loss-of-Function Promotes Renal Cancer Branched Evolution through Replication Stress and Impaired DNA Repair. Oncogene.

[53] Liu J., Hanavan P.D., Kras K., Ruiz Y.W., Castle E.P., Lake D.F., Chen X., O’Brien D., Luo H., Robertson K.D. (2018). Loss of SETD2 Induces a Metabolic Switch in Renal Cell Carcinoma Cell Lines toward Enhanced Oxidative Phosphorylation. J. Proteome Res..

[54] Napolitano L., Orecchia L., Giulioni C., Carbonara U., Tavella G., Lizzio L., Fimognari D., De Palma A., Gheza A., Grosso A.A. (2022). The Role of miRNA in the Management of Localized and Advanced Renal Masses, a Narrative Review of the Literature. Appl. Sci..

[55] Chaumeil M.M., Bankson J.A., Brindle K.M., Epstein S., Gallagher F.A., Grashei M., Guglielmetti C., Kaggie J.D., Keshari K.R., Knecht S. (2024). New Horizons in Hyperpolarized ^13^C MRI. Mol. Imaging Biol..

[56] Gallagher F.A., Woitek R., McLean M.A., Gill A.B., Manzano Garcia R., Provenzano E., Riemer F., Kaggie J., Chhabra A., Ursprung S. (2020). Imaging Breast Cancer Using Hyperpolarized Carbon-13 MRI. Proc. Natl. Acad. Sci. USA.

[57] Chen H., Autry A.W., Brender J.R., Kishimoto S., Krishna M.C., Vareth M., Bok R.A., Reed G.D., Carvajal L., Gordon J.W. (2020). Tensor Image Enhancement and Optimal Multichannel Receiver Combination Analyses for Human Hyperpolarized ^13^ C MRSI. Magn. Reason. Med..

[58] Khegai O., Schulte R.F., Janich M.A., Menzel M.I., Farrell E., Otto A.M., Ardenkjaer-Larsen J.H., Glaser S.J., Haase A., Schwaiger M. (2014). Apparent Rate Constant Mapping Using Hyperpolarized [1–13C]Pyruvate. NMR Biomed..

[59] Iorio F., Knijnenburg T.A., Vis D.J., Bignell G.R., Menden M.P., Schubert M., Aben N., Gonçalves E., Barthorpe S., Lightfoot H. (2016). A Landscape of Pharmacogenomic Interactions in Cancer. Cell.

[60] Dobin A., Davis C.A., Schlesinger F., Drenkow J., Zaleski C., Jha S., Batut P., Chaisson M., Gingeras T.R. (2013). STAR: Ultrafast Universal RNA-Seq Aligner. Bioinformatics.

[61] Anders S., Pyl P.T., Huber W. (2015). HTSeq--a Python Framework to Work with High-Throughput Sequencing Data. Bioinformatics.

[62] Love M., Ahlmann-Eltze C., Forbes K., Anders S., Huber W., FP7 R.E., NHGRI N. CZI DESeq2: Differential Gene Expression Analysis Based on the Negative Binomial Distribution 2022. https://github.com/mikelove/DESeq2.

[63] Liu Y., Li G. (2023). Empowering Biologists to Decode Omics Data: The Genekitr R Package and Web Server. BMC Bioinform..

[64] Young M.D., Mitchell T.J., Vieira Braga F.A., Tran M.G.B., Stewart B.J., Ferdinand J.R., Collord G., Botting R.A., Popescu D.-M., Loudon K.W. (2018). Single-Cell Transcriptomes from Human Kidneys Reveal the Cellular Identity of Renal Tumors. Science.

[65] Chu T., Wang Z., Pe’er D., Danko C.G. (2022). Cell Type and Gene Expression Deconvolution with BayesPrism Enables Bayesian Integrative Analysis across Bulk and Single-Cell RNA Sequencing in Oncology. Nat. Cancer.

[66] Li H., Durbin R. (2009). Fast and Accurate Short Read Alignment with Burrows–Wheeler Transform. Bioinformatics.

[67] Jones D., Raine K.M., Davies H., Tarpey P.S., Butler A.P., Teague J.W., Nik-Zainal S., Campbell P.J. (2016). cgpCaVEManWrapper: Simple Execution of CaVEMan in Order to Detect Somatic Single Nucleotide Variants in NGS Data. Curr. Protoc. Bioinform..

[68] Raine K.M., Hinton J., Butler A.P., Teague J.W., Davies H., Tarpey P., Nik-Zainal S., Campbell P.J. (2015). cgpPindel: Identifying Somatically Acquired Insertion and Deletion Events from Paired End Sequencing. Curr. Protoc. Bioinform..

[69] Van Loo P., Nordgard S.H., Lingjærde O.C., Russnes H.G., Rye I.H., Sun W., Weigman V.J., Marynen P., Zetterberg A., Naume B. (2010). Allele-Specific Copy Number Analysis of Tumors. Proc. Natl. Acad. Sci. USA.

[70] GitHub—Cancerit/alleleCount: Support Code for NGS Copy Number Algorithms. Takes a File of Locations and a [Cr|b]Am File and Generates a Count of Coverage of Each Allele [ACGT] at That Location (Given Any Filter Settings). https://github.com/cancerit/alleleCount.

[71] Bolli N., Avet-Loiseau H., Wedge D.C., Van Loo P., Alexandrov L.B., Martincorena I., Dawson K.J., Iorio F., Nik-Zainal S., Bignell G.R. (2014). Heterogeneity of Genomic Evolution and Mutational Profiles in Multiple Myeloma. Nat. Commun..

